# *Planoplatessa* gen. nov.—A New, Neglected Monoraphid Diatom Genus with a Cavum

**DOI:** 10.3390/plants11172314

**Published:** 2022-09-03

**Authors:** Maxim S. Kulikovskiy, Anton M. Glushchenko, Irina V. Kuznetsova, John Patrick Kociolek

**Affiliations:** 1 K.A. Timiryazev Institute of Plant Physiology RAS, IPP RAS, 35 Botanicheskaya St., 127276 Moscow, Russia; 2 Museum of Natural History, Henderson Building, 15th and Broadway, Boulder, CO 80309, USA

**Keywords:** diatoms, *Planoplatessa*, *Platessa*, new genus, morphology, Mongolia, Lake Khövsgöl

## Abstract

A new monoraphid diatom genus, *Planoplatessa* gen. nov., is described on the basis of a detailed morphological investigation using light and scanning electron microscopy. We transferred *Achnanthes joursacensis* Héribaud, a species described from fossil deposits in France, to our new genus. We investigated material from Mongolia from Recent populations. This taxon is known from fossils to Recent across the Holarctic. *Achnanthes joursacensis* was previously transferred to the genera *Planothidium* and *Platessa*, but the morphology of *A. joursacensis* does not share all morphological features with these two genera. We discuss important morphological features for the delimitation of monoraphid genera based on careful morphological and molecular investigations we presented previously. *Planoplatessa* gen. nov. as a genus is characterized by having uniseriate striae on both the raphe and the rapheless valves, a cavum in rapheless valves only, and straight distal raphe ends on the valve face.

## 1. Introduction

About 16 freshwater monoraphid diatom genera previously associated with the genus *Achnanthes* J.B.M. Bory de Saint-Vincent 1822 [1] are currently recognized: *Achnanthidium* Kützing 1844 [2], *Eucocconeis* P.T. Cleve 1895 [3], *Psammothidium* Bukhtiyarova & Round 1996 [4], *Karayevia* Round & Bukhtiyarova 1996 [5], *Rossithidium* Round & Bukhtiyarova 1996 [5], *Lemnicola* Round & Basson 1997 [6], *Crenotia* Wojtal 2013 [7], *Trifonovia* Kulikovskiy & Lange-Bertalot 2012 [8], *Gololobovia Gliwiczia* Kulikovskiy, Lange-Bertalot & Witkowski 2013 [9], *Skabitschewskia* Kulikovskiy & Lange-Bertalot 2015 [10], *Platessa* Lange-Bertalot 2004 [11], *Planothidium* Round & Bukhtiyarova 1996, Kulikovskiy, Glushchenko, Genkal & Kociolek 2020 [12], *Gogorevia* Kulikovskiy, Glushchenko, Maltsev & Kociolek 2020 [13], *Platebaikalia* Kulikovskiy, Glushchenko, Genkal & Kociolek 2020 [14], and *Gomphothidium* Kociolek et al. 2022 [15].

Of the above genera, only *Gliwiczia*, *Planothidium*, and *Skabitschewskia* have a very interesting morphological feature that is visible under a light microscope known as a horseshoe-shaped area [10]. The morphology of this horseshoe-like structure as seen with scanning electron microscopy includes a rimmed depression or sinus and hood or cavum (see description in [16]). Interestingly, species that possess a sinus and cavum, and those species where these features are weakly expressed have been shown to be closely related to one another and included in the genus *Planothidium* [16]. According to molecular data, these species can be recognized as three distinct clades: (a) species with a sinus, (b) species with a cavum, or (c) species without these features [16]. The genus *Skabitschewskia* is characterized by having only a cavum on rapheless valves in all known species, but this genus differs from *Planothidium* by striae morphology [10]. Striae in *Planothidium* are multiseriate on both valves, whereas, in *Skabitschewskia*, striae are uniseriate on raphe valves and biseriate on rapheless valves [10]. In the genus *Gliwiczia*, a cavum is present on both valves, and the striae are also uniseriate on both valves [9].

It is evident from the data discussed above that presence and number of horseshoe-like structures and morphology of striae are important features for the delimitation of genera between freshwater monoraphid taxa. The morphology of striae between monoraphid genera was comprehensively discussed by us during the description of the new genus *Platebaikalia* [14]. Freshwater monoraphid genera have different combinations of striae on raphe and rapheless valves that can include the presence of uniseriate, biseriate, or multiseriate striae. On the basis of this morphological feature, we prepared a revision of the genus *Platessa,* a genus that was described on the basis of species with biseriate striae in both valves and the absence of horseshoe-shaped structure [17]. After this description, many species were transferred to this genus, and *Platessa* soon became a catch-all taxon [14]. We described eight groups within the genus *Platessa*, and some of these groups have subsequently been described as new genera [14]. One of the eight groups of *Platessa* species includes the very interesting species *Platessa joursacense* (Héribaud) Chudaev which has uniseriate striae on both valves, but this species possesses a cavum on the rapheless valves [18].

*P. joursacense* was firstly described as *Achnanthes joursacensis* Héribaud 1903 [19] and for a long time was a neglected taxon. The generic position of this species has been interpreted differently by several authors. This species was included in the genus *Planothidum* as *Planothidium joursacense* (Héribaud) Lange-Bertalot 1999 [20] and later in *Platessa* as *Platessa joursacensis* (Héribaud) Chudaev [18]. The reason for this taxonomical instability is, on the one hand, the presence of cavum being a feature considered typical for *Planothidum* and, on the other hand, uniseriate striae in both valves not being typical for *Planothidum*. The presence of distal raphe ends that terminate on the valve face and straight is typical for *Platessa*. For a long time, this species was neglected due to its rarity and limited (only Holarctic) distribution [11]. Morphology of the raphe and rapheless valves was first investigated by Chudaev et al. [18].

The aim of this publication is to provide additional morphological evidence for the monoraphid diatom, *Achnanthes joursacensis* Héribaud 1903 and, on the basis of the results of this study, to describe the genus *Planoplatessa* gen. nov.

## 2. Results

### Planoplatessa Kulikovskiy, Glushchenko & Kociolek gen. nov.

Type species (designated here): *Planoplatessa joursacensis* (Héribaud) Kulikovskiy, Glushchenko & Kociolek comb. nov.

### Description

**Figure 1:**
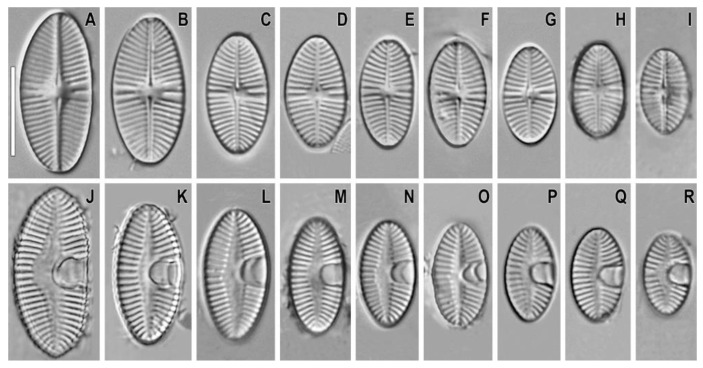
*Planoplatessa joursacensis* (Héribaud) Kulikovskiy, Glushchenko & Kociolek comb. nov. Slide no. 02605. Light microscopy, differential interference contrast, size diminution series. (**A**–**I**). Raphe valves. (**J**–**R**). Rapheless valves. Scale bar = 10 μm.

Light microscopy (LM), raphe valves (Figure 1A–I). Valves broadly elliptic with broadly rounded ends. Length 9.3–19.3 µm, width 5.5–8.4 µm. Raphe straight and filiform. Central area small and roundish. Axial area moderately narrow, almost linear. Striae 17–19 in 10 µm. Striae radiate at the center of the valve; very strongly radiate and curved toward the ends.

LM, rapheless valves (Figure 1J–R). Axial area narrowly lanceolate. Striae spaced slightly wider than in raphe valves. Visible horseshoe-shaped structure is present.

**Figure 2:**
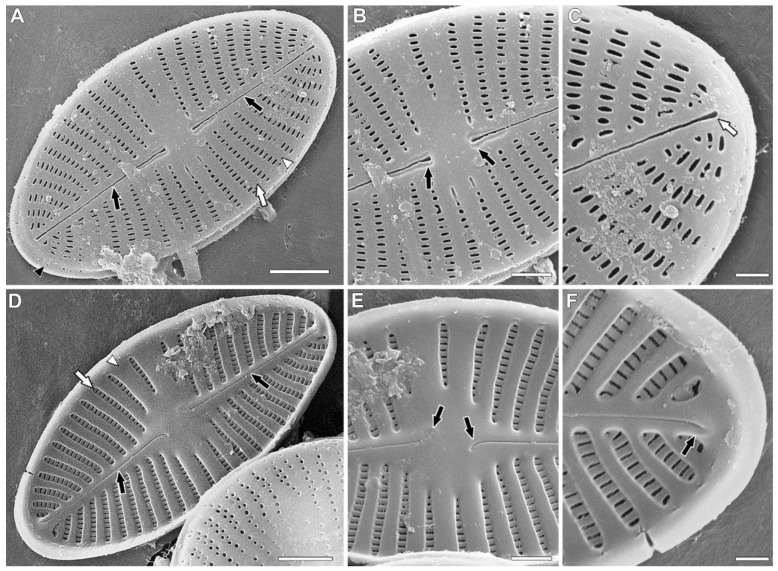
*Planoplatessa joursacensis* (Héribaud) Kulikovskiy, Glushchenko & Kociolek comb. nov. Scanning electron microscopy. Raphe valves. (**A**). The entire valve, external view. Black arrows show the filiform and straight raphe. White arrow shows the uniseriate stria. Black arrowhead shows the valve mantle. White arrowhead shows the interstria. (**B**). Central area, external view. Black arrows show the central raphe ends. (**C**). Valve end, external view. White arrow shows the distal raphe end. (**D**). The whole valve, internal views. Black arrows show the filiform and straight raphe. White arrow shows the uniseriate stria. White arrowhead shows the interstria. (**E**). Central area, internal view. Black arrows show the deflected in opposite directions central raphe ends. (**F**). Valve end, internal view. Black arrow shows the helictoglossa. Scale bar (**A**,**D**) = 2 μm; (**B**,**E**) = 1 μm; (**C**,**F**) = 0.5 μm.

Scanning electron microscopy (SEM), raphe valves (Figure 2A–F). Striae uniseriate (Figure 2A,D, white arrows). Raphe filiform and straight (Figure 2A, black arrows). Distal raphe ends straight and present on valve face outside (Figure 2C, white arrow). Central raphe ends straight and tear-shaped outside (Figure 2B, black arrows). Mantle is short, with two small areolae present in prolongation of every striae (Figure 2A, black arrowhead). Interstriae are wider than striae inside and outside (Figure 2A,D, white arrowhead). Inside, distal raphe ends in smaller helictoglossae and curved in opposite direction (Figure 2F, black arrow). Central raphe ends are slim and curved to different sides (Figure 2E, black arrows). Distal and central raphe ends are curved opposite to another. Areolae ≈ 60 in 10 µm.

**Figure 3:**
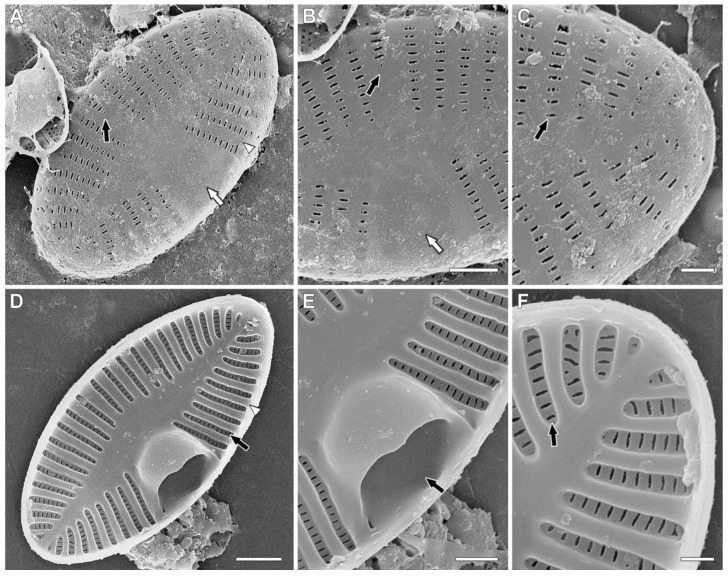
*Planoplatessa joursacensis* (Héribaud) Kulikovskiy, Glushchenko & Kociolek comb. nov. Scanning electron microscopy. Rapheless valves. (**A**). The entire valve, external view. Black arrow shows the shortened central stria. White arrow shows the central area. White arrowhead shows the interstria. (**B**). Central area, external view. Black arrow shows the shortened central stria. White arrow shows the central area. (**C**). Valve end, external view. Black arrow shows the uniseriate stria. Black (**D**). The whole valve, internal view. Black arrow shows the uniseriate stria, situated in deep alveola. White arrowhead shows the interstria. (**E**). Internal view. Black arrow shows the cavum. (**F**). Valve end, internal views. Black arrow shows the uniseriate stria, situated in deep alveola. Scale bar (**A**,**D**) = 2 μm; (**B**,**E**) = 1 μm; (**C**,**F**) = 0.5 μm.

SEM, rapheless valves (Figure 3A–F). Striae uniseriate (Figure 3D, black arrow). Interstriae are wider than striae. Areolae covered by silica membrane internally [18].

Externally, central area large and flat on one side of valve (Figure 3A,B, white arrows); in another part, all striae are normally present or one central stria is slightly shorter (Figure 3A,B, black arrows). Internally, a large cavum is present (Figure 3E, black arrow). Striae are situated in deep alveoli (Figure 3D,F, black arrows).

### Etymology

Combining epithet refers to the similarity with two monoraphid diatom genera, Platessa and Planothidium.

### New Combination

*Planoplatessa joursacensis* (Héribaud) Kulikovskiy, Glushchenko & Kociolek comb. nov.

Basionym: *Achnanthes joursacensis* Héribaud 1903, in *Les Diatomées Fossiles d’Auvergne Paris*: 5; pl. 11, Figures 26 and 27.

≡ *Planothidium joursacense* (Héribaud) Lange-Bertalot 1999, Iconographia Diatomologica 6: 283. ≡ *Platessa joursacensis* (Héribaud) Chudaev 2015, Novosti Sist. Nizsh. Rast. 49: 112–113.

## 3. Discussion

*Achnanthes joursacensis* was described by Héribaud from fossil material (Upper Miocene or Pontus) near Joursac Village (currently Commune), from the southern part of Central France [19]. Subsequently, this taxon was found in sediments of different Epochs of the Neogene Period, including Miocene [21] and Pliocene [22,23], as well as from the Quaternary Period, both Pleistocene [24] and Recent in Europe [25], Russia, Moscow Region [18], Russian Far East [26], Mongolia (this investigation), Japan [27], and USA [28]. The lectotype of the species was investigated and illustrated by Krammer and Lange-Bertalot, taf. 47, Figures 7–9 [29]. This taxon prefers alkaline, meso- to eutrophic lakes and lake outlets [25]. We found this taxon in weakly alkaline (pH = 8.7) Lake Khövsgöl (Mongolia) with an electrical conductivity of 236 μS·cm^−1^.

All findings of this species, with both LM and SEM, correspond to observations of the lectotype investigated previously. This taxon is characterized by uniseriate striae on both valves and the presence of a cavum on rapheless valves; external distal raphe ends are straight and placed on the valve face and do not extend onto the mantle [18,29]. This combination of morphological features makes this species easily recognizable. Populations of this species were found in the Holarctic, both fossil to Recent [18].

The presence of a cavum was a good reason to transfer this species to the genus *Planothidium* [20]. However, *Planothidium* as a genus is also characterized by having multiseriate striae on both valves. *Planoplatessa joursacensis* comb. nov., however, only has uniseriate striae [10]. External distal raphe ends extend onto the valve mantle in *Planothidium*; however, in *Planoplatessa joursacensis* comb. nov., they are straight and only occur on the valve face. As discussed above, *Planothidium* as a genus can be divided into three groups, one with cavum, a second with sinus, and a third without a cavum and sinus. However, all the taxa in these groups of *Planothidium* have multiseriate striae. *Skabitschewskia* is another genus with the presence of a cavum in rapheless valves. However, *Skabitschewskia* is characterized by uniseriate striae on the raphe valves and biseriate striae on the rapheless valves [10]. The genus *Gliwiczia* is characterized by the presence of a cavum and uniseriate striae on both valves similar to the situation in *Planoplatessa* gen. nov. However, *Gliwiczia* has a cavum on both raphe and rapheless valves, a situation that is unique among monoraphid genera.

Genera such as *Achnanthidium*, *Eucocconeis*, *Psammothidium*, *Trifonovia*, *Gololobovia*, and *Gogorevia* are characterized by having uniseriate striae on both valves [8,10,12,13]. However, these genera do not have a cavum, a feature that is typical for *Planoplatessa* gen. nov. All these genera are very easy to distinguish from *Planoplatessa* gen. nov. by a combination of morphological features such as valve shape, presence or absence of sternum and stauros internally and externally, and morphology of distal and central raphe ends (see Table 1). Combinations of stria morphology with the presence or absence of a cavum and stauros are important taxonomical features that were shown with molecular data to help diagnose freshwater monoraphid genera such as *Achnanthidium*, *Psammothidium*, *Gogorevia*, *Karayevia*, *Planothidium*, and *Lemnicola* [13,16,30,31,32,33,34].

*Planoplatessa* gen. nov. as a genus is distinguished from other known monoraphid genera. Previous transfer of *A*. *joursacensis* to the genus *Platessa* was based on some morphological features shared between them, especially external distal raphe ends being straight and extending onto the valve face only [18]. Transfer of taxa that were divided on some morphological features between similar genera to different genera is normal practice. We discussed the same situation with the previous transfer of *Achnanthes exigua* Grunow 1880 to the genera *Achnanthidium* and *Lemnicola* [13]. However, our molecular investigation of species from this complex showed that a new genus was required, which we described as *Gogorevia*. Future research combining morphological and molecular datasets will be necessary to further resolve relationship of the other freshwater monoraphid genera and help to identify features that allow us to not only recognize and distinguish the genera, but also diagnose monophyletic taxa and create a natural classification for them [35].

## 4. Materials and Methods

Samples from Mongolia were collected by M.S. Kulikovskiy on 21 July 2015 from the Khövsgöl Lake (50°59′22.8″ N, 100°42′30.4″ E; pH = 8.7; T = 11.5 °C; conductivity = 236 μS·cm^−1^), directly sampling benthos and designated Mn 282.

The samples were boiled in concentrated hydrogen peroxide (≈37%) to dissolve organic matter. The samples were then washed with deionized water four times at 12 h intervals. After decanting and rinsing with up to 100 mL of deionized water, the suspension was spread onto coverslips and left to dry at room temperature. Permanent diatom slides were mounted in Naphrax^®^. The slide was designated no. 02605. Light microscopic (LM) observations were performed using a Zeiss Scope A1 microscope equipped with an oil immersion objective (100×, n.a. 1.4, differential interference contrast (DIC)) and Zeiss AxioCam ERc 5 s camera. For scanning electron microscopy (SEM), parts of the suspensions were fixed on aluminum stubs after air-drying. The stubs were sputter-coated with 50 nm of gold. The valve ultrastructure was examined by means of a JSM-6510LV scanning electron microscope (Institute for Biology of Inland Waters RAS, Borok, Russia).

## 5. Conclusions

New insights into character combinations found in freshwater monoraphid diatoms are demonstrating key features used to diagnose genera. An easily recognizable feature in both light and scanning electron microscopy is a cavum, a hollow covering found in the valve interior of certain monoraphid genera. We can now recognize four monoraphid genera that possess a cavum: *Planothidium, Skabitschewskia, Gliwiczia*, and *Planoplatessa* gen. nov. These genera are recognizable from one another on the basis of whether there is a single cavum per frustule or two (found in *Gliwiczia* only), multiseriate versus uniseriate striae, and whether the external distal raphe ends extend onto the valve mantle or are restricted to the valve face. While we expect these groups to constitute a monophyletic lineage, formal analyses based on morphology and molecules will be needed to verify taxon relationships and monophyly.

## Figures and Tables

**Table 1:** Comparison of different freshwater monoraphid diatom genera with cavum.

	*Planoplatessa* gen. nov.	*Skabitschewskia*	*Planothidium*	*Gliwiczia*
Type species	*P. joursacensis* (Héribaud) Kulikovskiy, Glushchenko & Kociolek comb. nov.	*S. dispersipunctata* Kulikovskiy & Lange-Bertalot 2015	*P. lanceolatum* (Brébisson ex Kützing) Lange-Bertalot 1999	*G. tenuis* Kulikovskiy, Lange-Bertalot & Witkowski 2013
Striae in raphe valves (RV)	Uniseriate	Uniseriate	Multiseriate	Uniseriate
Striae in rapheless valves (RLV)	Uniseriate	Biseriate	Multiseriate	Uniseriate
Interstriae in RV externally	Flat, wider than striae	Flat, equal to striae	Flat, less than striae	Flat, slightly broader than striae
Interstriae in RV internally	Very prominent on striae, broader than striae	Prominent, equal to or broader than striae	Prominent, narrower than striae	Slightly prominent, slightly broader than striae
Interstriae in RLV externally	Flat, broader than striae	Prominent, equal to or broader than striae	Flat, narrower than striae	Flat, equal to or broader than striae
Interstriae in RLV internally	Very prominent on striae, broader than striae	Very prominent (rib-like), connected with sternum, close areolae by silica layer (alveoli), narrower than striae; in some species with reduced striae the interstriae are longer and broader	Evidently raised and narrower than striae	Slightly raised, narrower than striae
Pore occlusions	Silica membrane	Silica membrane	Hymenes	Silica membrane, below the occlusion a pair of foramina lips
Distal raphe ends externally	Straight, on valve face near mantle	Straight or slightly curved on valve face or extending slightly onto valve mantle; turned in opposite directions	Smoothly curved to the same direction and extending onto valve mantle	Straight and extending slightly onto mantle, slightly deflected in opposite directions
Distal raphe ends internally	In small helictoglossae; curved in the opposite directions	In small helictoglossae; turned in different directions	In small helictoglossae; turned in different directions	Helictoglossae almost undeveloped; slightly turned in opposite directions
Central raphe ends externally	Tear-shaped, straight	Tear-shaped, straight	Tear-shaped, turned to the same direction and opposite to distal ends	Tear-shaped, straight
Central raphe ends internally	Linear, straight, curved in opposite directions	Straight; turned to the different direction	Straight; turned in different directions	Straight; evidently turned in different directions
Axial area in RV, externally	Flat, narrow and linear	Narrow and linear, sternum	Narrow and linear	Narrow and linear, sternum
Axial area in RV, internally	Narrow and straight, sternum evident and elevated	Sternum well-developed, narrow and linear	Sternum well-developed, narrow and linear	Sternum well-developed, narrow and linear
Axial area in RLV, externally	Flat, lanceolate	Narrow and linear or slightly wider to central area, in many species deep on valve face	Narrow and linear or slightly wider to central area, deep on valve face	Narrow and wider to central area, almost rhombic
Axial area in RLV, internally	Flat, lanceolate	Narrow lanceolate, raised on valve face in middle	Narrow and linear or wider to central area, sternum well-developed	Broad rhombic sternum
Central area in RV externally	Small, circle	Circle or bowtie-shaped, flat or slightly raised in center	Striae either not shortened or 1–2 slightly shortened striae forming a small circular area	Stauros elevated, central nodule evidently raised
Central area in RV internally	Small, circle	Circle or bowtie-shaped, raised in center	Striae shortened or 1–2 slightly shortened striae forming a small circular area	Stauros strongly elevated with cavum on one side
Central area in RLV externally	Flat and large trapezium in one side	Flat, fascia in one cavum side of valve	Flat hyaline area on one side, striae either not shortened or 1–2 slightly shortened striae forming a small circular area	Stauros elevated
Central area in RLV internally	Large cavum in one side	Cavum	Sinus on one side, striae either not shortened or 1–2 slightly shortened striae forming a small circular area	Stauros strongly elevated with cavum on one side
References	This investigation, [18]	[10]	[5,30]	[10], own data

## Data Availability

Not applicable.

## Abbreviations

LM—light microscopy; SEM—scanning electron microscopy; RV—raphe valves; RLV—rapheless valves.
